# Advances in Human B Cell Phenotypic Profiling

**DOI:** 10.3389/fimmu.2012.00302

**Published:** 2012-10-10

**Authors:** Denise A. Kaminski, Chungwen Wei, Yu Qian, Alexander F. Rosenberg, Ignacio Sanz

**Affiliations:** ^1^Division of Allergy, Immunology, and Rheumatology, Department of Medicine, University of RochesterRochester, NY, USA; ^2^Department of Pathology, University of Texas Southwestern Medical CenterDallas, TX, USA

**Keywords:** B lymphocyte, human, flow cytometry, data management, autoimmunity, data clustering

## Abstract

To advance our understanding and treatment of disease, research immunologists have been called-upon to place more centralized emphasis on impactful human studies. Such endeavors will inevitably require large-scale study execution and data management regulation (“Big Biology”), necessitating standardized and reliable metrics of immune status and function. A well-known example setting this large-scale effort in-motion is identifying correlations between eventual disease outcome and T lymphocyte phenotype in large HIV-patient cohorts using multiparameter flow cytometry. However, infection, immunodeficiency, and autoimmunity are also characterized by correlative and functional contributions of B lymphocytes, which to-date have received much less attention in the human Big Biology enterprise. Here, we review progress in human B cell phenotyping, analysis, and bioinformatics tools that constitute valuable resources for the B cell research community to effectively join in this effort.

## Introduction

Like other areas of immunology, much of our understanding of B cell biology is based on laboratory mouse studies. Despite bountiful experimental capabilities, mouse systems are sub-optimal models of normal human B cell function or of genuine human disease, as demonstrated by failed interspecies transfer of therapeutic protocols in clinical trials (Davis, 2008). However, findings in mice can motivate informative investigation in humans, provided that we acknowledge key differences: (1) humans are largely outbred, (2) they live in diverse environments that researchers have no control over and have limited abilities to monitor, and (3) most human tissues are inaccessible, and accessible lymphoid tissues may not necessarily drain the area of interest. For these latter reasons, peripheral blood mononuclear cell (PBMC) preparations are the most commonly analyzed cell source in human immunology research. With the exception of blatant systemic sepsis, PBMC should be viewed as a sentinel population that indirectly informs us of immunological activity occurring elsewhere. Similar to observing diversely dressed people on a daily commute, we can only infer the origins, occupations, accomplishments, and capabilities of PBMC based on previous correlative evidence. Such correlations are only meaningfully applied to generalized conclusions about humans if we have large numbers of subjects and a resource base (labor, materials, instrumentation, and data management infrastructure) enabling utilization of human samples to their fullest extent.

Besides our devoted curiosity, an important rationale for investing in human B cell research is based on the observation that B cell-depletion can result in clinical improvement in autoimmune diseases, including systemic lupus erythematosus (SLE), rheumatoid arthritis, primary Sjögren’s Syndrome, type 1 diabetes, and neurological disorders, such as multiple sclerosis (Anolik et al., 2004, 2007; Leandro et al., 2006; Pranzatelli et al., 2006; Steinfeld et al., 2006; Dass et al., 2008; Hauser et al., 2008; Jacob et al., 2008; Roll et al., 2008; Moller et al., 2009; Nakou et al., 2009; Pescovitz et al., 2009; Meijer et al., 2010; Iwata et al., 2011a). Importantly, only some, but not all effects attributed to B cells are autoantibody-mediated (Anolik et al., 2004; Sanz, 2011), an outcome also observed in mouse models (Chan and Shlomchik, 1998). However, it is not entirely clear exactly which antibody-independent B cell functions (antigen presentation, cytokine production, or lymphoid structure development; Lund and Randall, 2010; Marcus et al., 2011), when absent, account for the therapeutic benefit. Understanding which B cell subsets are responsible for which functions in these diseases, in addition to identifying how a “signature” profile of an individual subject’s collection of subsets may correlate with disease outcome could eventually allow greater optimization of targeted therapies (Anolik et al., 2009; Sanz and Lee, 2010).

## B Cell Subsets in Healthy Humans

Newly formed transitional B cells with an intact B cell receptor (BCR) emigrate from bone marrow into peripheral circulation and secondary lymphoid organs (Anolik et al., 2009). After cessation of B cell-depletion therapy, the earliest transitional B cells reconstituting the human periphery are CD10^+^CD38^high^CD24^high^, and due to an inactive mitochondrial transporter protein, can also retain reagent dyes such as Mitotracker Green^®^ (MTG; Wirths and Lanzavecchia, 2005; Blair et al., 2010). Transitional B cells are further subdivided into “T1” and “T2”, as well as CD10^neg^ MTG-retaining “T3” cells, based on incrementally lower expression of CD24 and CD38, inversely correlating with time of reconstitution (Palanichamy et al., 2009). *In vitro*, these cells are capable of stepwise differentiation into mature-naïve-phenotype B cells, although their obligate precursor-progeny relationships *in vivo* await confirmation. Similar to T3, mature-naïve B cells are CD10^neg^CD38^low/neg^, but extrude MTG (Wirths and Lanzavecchia, 2005). Compared with transitional-phenotype B cells, the mature-naïve fraction survives longer in culture, and divides more readily upon BCR engagement by cognate antigen (Wirths and Lanzavecchia, 2005).

B cell receptor engagement and B cell costimulation through cell-surface CD19 and CD21 on mature-naïve B cells can stimulate activation and differentiation (Harwood and Batista, 2010; Kurosaki, 2011), which ideally contribute to eliminating invading organisms. Early cell-surface changes associated with B cell activation include the up-regulation of CD40, CD80, CD86, and CD69. More than half of naïve-phenotype B cells express the Fcε receptor CD23 (Kikutani et al., 1986; Kumagai et al., 1989; Klein et al., 1998; Tangye et al., 1998; Quach et al., 2011). Although CD23 can be up-regulated after *in vitro* stimulation (Pelton et al., 1991), tonsilar B cells showing evidence of recent activation lack CD23 expression (Kolar et al., 2007). Whether this differential expression reflects distinct subsets or a consequence of *in vivo* stimuli is unknown.

Activated B cells receiving T cell help in secondary lymphoid tissue follicles can become germinal center (GC) B cells. GC B cells proliferate and can class-switch the BCR constant region from IgM/IgD to IgG, IgA, or IgE (Pascual et al., 1994). Additionally, the Ig V region genes of GC B cells can undergo somatic hypermutation to change the affinity of the encoded BCR for its cognate antigen, thus allowing subsequent antigen-driven selection and clonal expansion of high-affinity B cells. When peripheral tolerance is intact, these B cells are mostly not self-reactive, and differentiate into antibody-secreting plasma cells or into memory B cells that can rapidly respond to a subsequent encounter with an invading organism.

Human GC B cells, *in vitro*-activated naïve B cells, and those with specific and rapid recall responses to previously encountered antigens (memory) express cell-surface CD27. B cell CD27 expression correlates with greater cell size (Agematsu et al., 1997; Wirths and Lanzavecchia, 2005), proliferative capacity (Tangye et al., 2003a,b; Macallan et al., 2005; Good et al., 2009), antigen presentation capacity (Good et al., 2009), and differentiation into antibody-secreting cells (ASC; Tangye et al., 2003b). Because ASC in circulation are at low frequencies, because few circulating CD27^+^ B cells show evidence of on-going proliferation, and because GC are generally confined to lymphoid tissues, most CD27^+^ B cells in the peripheral pool of healthy humans are typically considered “memory B cells.” Consistent with this designation, the Ig V regions of CD27^+^ B cells tend to have somatic hypermutations with DNA sequence characteristics of antigen selection, regardless of whether the cells are IgD^neg^ [mostly class-switched memory (“switched memory”)] or are IgD/IgM^+^ (“non-switched memory”; Pascual et al., 1994; Dunn-Walters et al., 1995; Klein et al., 1998; Tiller et al., 2007). Reports differ on whether the non-switched memory and switched memory compartments share an Ig Vh repertoire (Odendahl et al., 2000; Weller et al., 2008; Seifert and Kuppers, 2009; Wu et al., 2010), making it uncertain whether or not the latter compartment derives from the former. Deep sequencing and clonality analysis showed that non-switched memory cells have a lower frequency of SHM than switched memory, and failed to find evidence that non-switched memory cells were clonally related to the switched memory pool (Wu et al., 2011). Although functional mouse studies show that at least some of the IgM memory pool maintains long-term IgG memory against protein antigen (Dogan et al., 2009; Pape et al., 2011), the human non-switched memory pool has characteristics not shared with the switched memory fraction. These characteristics include GC-independent somatic hypermutation (Weller et al., 2001), attributes of putative B1 B cells, regulatory B cells, and splenic marginal zone-like B cells that contribute carbohydrate-reactive antibody responses to encapsulated bacteria (Kruetzmann et al., 2003; Tsuiji et al., 2006; Wasserstrom et al., 2008; Griffin et al., 2011; Iwata et al., 2011b). Thus, the IgD^+^CD27^+^ pool is likely a composite of at least two, if not several functional subsets, possibly together with some precursors that will class-switch to feed the switched memory pool.

Some class-switched human B cells are IgD^neg^CD27^neg^ (“double-negative”) that are typically less than 5% of the CD19^+^ population in healthy subjects (Fecteau et al., 2006; Wei et al., 2007). mRNA analysis indicates that a minor population of this fraction expresses IgM without IgD (Wu et al., 2011). Both CD27^+^ (switched memory) and CD27^neg^ (double-negative) IgD^neg^ fractions can be stimulated to secrete immunoglobulin against tetanus toxin and influenza virus (Wirths and Lanzavecchia, 2005), suggesting involvement in prior vaccine responses. Apart from CD27, These two B cell fractions have similar surface phenotypes and have somatically mutated Ig V regions (Wirths and Lanzavecchia, 2005; Fecteau et al., 2006; Wei et al., 2007). However, fewer double-negative B cells have somatic mutations than switched memory B cells (Fecteau et al., 2006; Wei et al., 2007), suggesting that the former fraction is a mixture of both true memory B cells and something else, possibly transient effectors. Deep sequencing and clonality analysis suggest that double-negative B cells can become switched memory and *vice-versa* (Wu et al., 2011). Thus, the pools of memory B cells in circulation may go through distinct differentiation stages in which CD27 expression reversibly changes in class-switched B cells.

Appropriately activated B cells can differentiate into ASC, which help resolve primary infections and are also maintained for long-term protection (Fairfax et al., 2008; Oracki et al., 2010). Although rare in the blood of healthy, unchallenged individuals, PB ASC rapidly and transiently increase within 1 week after vaccination or infection (Blink et al., 2005; Odendahl et al., 2005; Gonzalez-Garcia et al., 2006; Wrammert et al., 2008, 2011; Blanchard-Rohner et al., 2009; Halliley et al., 2010; Lee et al., 2010, 2011; Qian et al., 2010; Li et al., 2012). The associated increase in serum antibody titer is sustained (Halliley et al., 2010), and can last for the lifetime of the individual (Slifka et al., 1998; Amanna et al., 2007). These sustained antibody levels are likely provided by long-lived ASC in other tissues, such as bone marrow, where they are abundant (Morell et al., 1970; McMillan et al., 1972; Slifka et al., 1998).

Most human blood CD19^+^CD27^high^CD38^high^ ASC are considered plasmablasts due to evidence suggesting on-going cell division (Odendahl et al., 2005; Wirths and Lanzavecchia, 2005; Gonzalez-Garcia et al., 2006; Halliley et al., 2010; Qian et al., 2010). Plasmablasts can be distinguished from plasma cells, a term ideally reserved for truly terminally differentiated ASC (Fairfax et al., 2008; Oracki et al., 2010). Plasma cell characteristics such as large size, little to no surface immunoglobulin, and non-proliferation correspond with expression of the adhesion molecule CD138 (syndecan-1) on CD38^high^ B cells (Smith et al., 1996; Wirths and Lanzavecchia, 2005; Gordon et al., 2008; Perry et al., 2008; Caraux et al., 2010; Di Niro et al., 2010). Expression of CD138 on more CD38^high^ B cells compared with CD38^int^ B cells suggests that at least some plasmablasts may be precursors of plasma cells (Arce et al., 2004; Fairfax et al., 2008; Oracki et al., 2010). Less than half of blood ASC express CD138, but nearly all bone marrow ASC express this molecule (Medina et al., 2002; Gonzalez-Garcia et al., 2006; Fairfax et al., 2008; Oracki et al., 2010). Whereas circulating plasmablasts may provide the transient boost seen in existing serum Ig levels after vaccination or acute infection, long-lived CD138^+^ bone marrow plasma cells are likely responsible for long-lived antigen-reactive serum antibody, because concurrently detectable circulating memory B cells are not required for long-lived specific antibody in the serum (McMillan et al., 1972; Slifka et al., 1998; Bernasconi et al., 2002; Mamani-Matsuda et al., 2008). Differential expression of human CD38, CD138, and other markers including HLA-DR and CD20 (Terstappen et al., 1990; Kantele et al., 1996; Lakew et al., 1997; Medina et al., 2002; Tangye et al., 2003b; Arce et al., 2004; Gonzalez-Garcia et al., 2006; Doria-Rose et al., 2009; Jacobi et al., 2010b) may alternatively or additionally represent ASC subsets that derive from independent differentiation pathways in blood, bone marrow, and possibly other tissues.

B cells that can impair or suppress immune reactions are referred to as “regulatory” B cells (Bregs; Mauri and Bosma, 2012; Kaminski and Sanz, 2013). Antibody-independent suppressive activity can be mediated by antigen presentation to promote mouse regulatory T cell development (Redfield et al., 2011; Tadmor et al., 2011), cytotoxicity (Jahrsdorfer et al., 2006), reducing human T cell division (Bouaziz et al., 2010), and secreting soluble factors that reduce expression of inflammatory cytokines by human and mouse T cells (Blair et al., 2010; Bouaziz et al., 2010; Iwata et al., 2011b; Ramgolam et al., 2011; Maseda et al., 2012). *In vivo*, mouse B cell IL10 can limit accumulation of neutrophils and NK cells in spleen, and bacteria-induced inflammatory foci in liver (Maseda et al., 2012). Similarly, the most well-studied suppressive mechanism in human B cells is IL10 production, which can only be detected after *ex vivo* stimulation (Yanaba et al., 2009; Bouaziz et al., 2010; Iwata et al., 2011b). Such stimulation includes innate-like (e.g., TLR) and helper T cell (CD40) signals alone or together with cognate antigen (Duddy et al., 2007; Yanaba et al., 2009; Bouaziz et al., 2010; Iwata et al., 2011b). IL10-competent B cells are considered to be a distinct functional subset or collection of subsets because their IL10 production occurs in a relatively polarized fashion in which co-expression of inflammatory cytokines is disfavored (Amel Kashipaz et al., 2003; Duddy et al., 2007; Yanaba et al., 2009). IL10 can be expressed by most commonly defined human B cell subsets, and conclusions differ about whether the “most efficient” Bregs are contained within the CD24^high^CD38^high^CD27^neg^ (Duddy et al., 2007; Blair et al., 2010; Bouaziz et al., 2010) or within the CD27^+^ B cell fractions (Blair et al., 2010; Bouaziz et al., 2010; Iwata et al., 2011b). The observed differences could result from methodological disparity or from biological variation and immunological circumstances among subject groups. In the context of B cell profiling, neither single nor combinations of cell-surface markers has definitively functioned as a surrogate for IL10 production or general regulatory capacity.

Our knowledge about human B cell subsets described above (summarized in Table 1) offers a wealth of possibilities for diagnostic profiling that could help optimize therapeutic protocols on a per-patient basis in the future. Getting to this point, however, will require validating and compiling our research findings with an infrastructure of data organization as well as standardization of how different cell populations are designated. Efforts to standardize leukocyte ontologies have been initiated to address NIAID’s data-sharing initiative, which aims to encourage the re-use and the re-analysis of data to maximize the public’s investment in biomedical research (Diehl et al., 2011; Kong et al., 2011; Smith and Scheuermann, 2011). Effective ontology systems can facilitate retrieval and integration of this data (Smith and Scheuermann, 2011). For example, the Human Studies Database Project has been established to form an informatics infrastructure for inter-institutional sharing (Sim et al., 2010). This includes the Bioinformatics Integration Support Contract, which was used to develop a web-based immunology database and analysis portal (ImmPort^1^; Kong et al., 2011). Assigning definitive nomenclature to B cell subsets will be an on-going challenge given that few, if any phenotypically defined populations are homogeneous in experience, function, etc. This effort will be best informed by a thorough understanding of B cell subset changes in the context of immune perturbation.

**Table 1:** **Surface-phenotype defined human peripheral B cell subsets**.

Name	Type	Phenotype	Markers to sub-fractionate	Ascribed functions	Biomarker potential
Transitional	T1	**IgD^+^CD27^neg^** CD10^+^CD24^high^ CD38^high^MTG^+^		Precursor to T2; IL10 production (?)	Early reconstitution ∞ SLE remission post-BCDT
	T2	**IgD^+^CD27^neg^** CD10^+^CD24^high/+^ CD38^high/+^MTG^+^		Precursor to T3; IL10 production (?)	↓ IL10 in SLE ↑ ∞ transplant tolerance ↑ in SS
	T3	**IgD^+^CD27^neg^** CD10^neg^CD24^+/low^ CD38^+/low^MTG^+^		Precursor to mature-naïve; IL10 production (?)	
Mature-naïve		**IgD^+^CD27^neg^** CD10^neg^CD24^+/low^ CD38^+/low^MTG^neg^	CD23, CD69, CD80, CD86	Precursor to GC, memory, and antibody-secreting cells	↑ activation in SLE
Memory	Double-negative	**IgD^neg^CD27^neg^**	CD21, CD24, CD95, CXCR3	Recall responses (and effector functions?)	↓ in SS ↑ ∞ autoAb and nephritis in SLE
	Non-switched	**IgD^+^CD27^+^**	CD1c, CD21, CD24	Immunoprotective self antibody (?); circulating MZ-like (?); regulatory (?)	↓ in SLE; inverse correlation with autoAb
	IgM-only	IgM^+^**IgD^neg^CD27^+^**	CD1c, CD21, CD24	Immunoprotective self antibody (?); circulating MZ-like (?); regulatory (?)	↑ in SS
	Switched	IgM^neg^ **IgD^neg^CD27^+^**	CD21, CD24, CD95, CXCR3	Pathogen protection; autoimmune pathology	↑ ∞ relapse in SLE and RA post-BCDT
					↓ ∞ poor vaccine response in elderly
Antibody-secreting cell	Plasmablast	**IgD^neg^CD27^high^** CD38^high^CD138^neg^	CD20, HLA-DR	Antibody secretion	↑ in SLE
	Plasma cell	**IgD^neg^CD27^high^** CD38^high^CD138^+^	CD20, HLA-DR	Antibody secretion	

*Markers in **bold** font indicate commonly defined “core” subsets; biomarker potential for T1 and T2 are merged as well plasmablast and plasma cell because they were not experimentally distinguished from each other*.

*MTG, mitotracker green; SLE, systemic lupus erythematosus; RA, rheumatoid arthritis; SS, Sjögren’s syndrome; BCDT, B cell-depletion therapy; autoAb, autoantibody levels*.

*∞, “correlates or positively associates with”; ↑, increased; ↓, decreased*.

## B Cell Alterations in Autoimmunity Suggest Possible Disease Signatures

Our understanding of human B cell function derives from comparisons between healthy individuals and those with particular immunological diseases, and among groups of patients having the same disease with different clinical outcomes. For example, human SLE is clinically heterogeneous (Bertsias et al., 2010), making treatment decisions challenging. Intriguingly, long-term remission in B cell-depleted SLE patients correlates with preferential early reconstitution of transitional-phenotype B cells after treatment is stopped (Anolik et al., 2007). Thus, these early B cell subsets either confer a protective effect and/or simply flourish (at the expense of memory-phenotype B cells) in the context of low disease activity. It remains to be determined whether intrinsic protective functions, such IL10 production (Blair et al., 2010) are directly responsible for this outcome in SLE. In organ transplant recipients, transitional-phenotype B cell deficiency and poor IL10 production by the remaining transitional-phenotype B cells correlate with immunosuppressive drug dependency, and in fact, transitional B cell frequencies can predict transplant tolerance (Newell et al., 2010). In primary Sjögren’s syndrome, the number of circulating transitional-phenotype B cells is higher than in controls, and modestly increases further several months after B cell-depletion therapy (Abdulahad et al., 2011). Whether this pattern corresponds with particular post-depletion clinical outcomes remains to be determined.

Systemic lupus erythematosus-patient B cells often show a heightened state of activation, including altered protein tyrosine phosphorylation patterns in IgD^+^CD27^neg^ (naïve-phenotype) and in total B cells (Liossis et al., 1996; Jenks and Sanz, 2009). SLE-patient B cells also show increased Ca^+2^ flux, cell size, proliferative capacity, antibody production, and activation-marker expression (Kumagai et al., 1989; Pelton et al., 1991; Liossis et al., 1996; Chang et al., 2008). Conflicting reports show lupus disease activity correlating with either low (Kumagai et al., 1989; Chang et al., 2008) or high (Pelton et al., 1991; Chang et al., 2008) CD23^+^ B cell frequencies. These results may reflect dissimilar B cell characterization or reflect subgroups of SLE patients with distinct CD23 B cell profiles. Our group recently described a poorly responsive subset of mature-naïve human B cells characterized as IgM^low^CD22^high^CD21^low^CD19^low^CD32b^low^, which are up to 15% autoreactive in healthy humans (Quach et al., 2011). Interestingly, such IgM^low^ cells from SLE patients have modestly lower levels of CD19 and CD22 compared with the IgM^low^ compartment of healthy subjects (Quach et al., 2011). Comprehensive investigation of B cell activation alterations, including those of this novel subset, will provide an opportunity to more clearly understand the mechanisms of breaking immunological tolerance in this and in other autoimmune diseases as well as potential phenotypic signatures that may predict their occurrence and outcomes.

Proliferating CD38^+^ GC structures in SLE-patient tonsils contain B cells with the self-reactive BCR idiotype, 9G4, which is typically excluded from GC in non-autoimmune control tonsils (Pugh-Bernard et al., 2001; Cappione et al., 2005). This peripheral checkpoint breach in SLE may be responsible for high 9G4^+^ serum autoantibody titers (Isenberg et al., 1993; van Vollenhoven et al., 1999) as well as IgG^+^9G4^+^ cells and 9G4^+^CD138^+^ plasma cells in SLE peripheral blood, which are otherwise rare in healthy controls (Cappione et al., 2005). Importantly, these observations demonstrate the 9G4 idiotype system to be a valuable device for tracking autoreactive B cells in lupus. Analysis of Sjögren’s patients showed proportionally increased CD38^high^IgD^+^ “GC founder” B cells in peripheral blood (Bohnhorst et al., 2001). Definitively determining to what degree these or GC-associated cells identified by other marker combinations in the blood can predict clinical outcomes will be advantageous for several B cell-mediated autoimmune diseases. This prediction is supported by the ablation of tonsilar GC structures in rheumatoid arthritis patients after TNF neutralization (Anolik et al., 2008), an effective therapy in many RA patients.

Unlike transitional-phenotype B cells, rapid reconstitution of CD27^+^ B cells correlates with clinical relapse of B cell-depleted SLE and rheumatoid arthritis patients (Leandro et al., 2006; Anolik et al., 2007; Roll et al., 2008; Moller et al., 2009; Nakou et al., 2009; Iwata et al., 2011a). Thus, CD27^+^ memory-phenotype B cells either contribute to and/or in other ways reflect autoimmune pathogenesis. This may also be true for the IgD^neg^CD27^neg^ double-negative B cell population whose variably higher frequencies in SLE correlate with high-titer autoantibody and nephritis incidence (Huang et al., 2002; Anolik et al., 2004; Wei et al., 2007). By contrast, double-negative B cells in primary Sjögren’s syndrome are proportionally reduced when measured as a fraction of CD27^neg^ B cells (Abdulahad et al., 2011). Reports differ on whether PB CD27^+^ B cells are lower (Abdulahad et al., 2011) or higher (Hansen et al., 2002) in primary Sjögren’s syndrome. This discrepancy may result from differences in B cell enumeration as well as differential occurrence of memory-phenotype B cell diversion to the parotid glands among different patients (Hansen et al., 2002). Analysis of a limited number of patients also suggest a possible correlation between high CD27^+^ B cells and lymphoma secondary to Sjögren’s (Hansen et al., 2002). Interestingly, blocking the activity of B cell Activation Factor of the TNF Family (BAFF) with biologics, including the recently FDA-approved recombinant monoclonal antibody Belimumab (Jefferson and Liscinsky, 2011; Sanz, 2011), effectively reduces numbers of transitional and mature-naïve B cells while increasing memory-phenotype B cells early after treatment in SLE and RA (Dall’Era et al., 2007; Tak et al., 2008; Wallace et al., 2009; Jacobi et al., 2010a). Clearly, further examination of these memory-phenotype compartments can yield informative metrics of disease status and may also provide further insight into the different roles of B cell subsets among different diseases. The utility of such profiling is unlikely limited to autoimmunity, because switched memory-phenotype B cell frequencies are reduced in older humans (≥65-years-old) compared with young adults (Frasca et al., 2010), statistically correlating with reduced systemic vaccine responses in the former group (Frasca et al., 2012).

Unlike the double-negative and switched memory populations, IgD^+^CD27^+^ non-switched memory B cells are proportionally reduced in active SLE (Wehr et al., 2004; Korganow et al., 2010; Rodriguez-Bayona et al., 2010), inversely correlating with autoantibody titers (Rodriguez-Bayona et al., 2010). Therefore, some of the non-switched memory/IgM memory B cells may play a protective role against pathology otherwise exacerbated by IgG memory. This conclusion is further supported by a stronger clinical correlation between serum 9G4^+^ IgG autoantibody compared with 9G4^+^ IgM (Bhat et al., 2002). Such a dichotomy of pathogenic and protective B cells has been observed in models of cardiovascular disease (Houtkamp et al., 2001; Sjoberg et al., 2009; Ait-Oufella et al., 2010; Kyaw et al., 2010). Curiously, in primary Sjögren’s syndrome, the proportion of IgM-only memory B cells (IgD^neg^IgM^+^ among CD27^+^ B cells) is increased compared with healthy controls (Abdulahad et al., 2011). This observation may suggest that the IgM-only population functions or is affected in this disease distinctly from the IgD^+^ non-switched memory compartment, that IgM-expressing memory cells participate differently in SLE compared with Sjögren’s syndrome, or both.

Protective contributions of regulatory B cells in mouse models of autoimmunity and allergy include B cell-intrinsic IL10 production, often corresponding with innate-like (MZ B and B1 B cell) phenotypes (Wolf et al., 1996; Zhou and Hansson, 1999; Fillatreau et al., 2002; Mizoguchi et al., 2002; Amu et al., 2010). B cell IL10 production can be decreased in induced autoimmune models but expanded in genetically predisposed autoimmune mice (Yanaba et al., 2009), and is also increased in subsets of patients with various autoimmune diseases, including those with rheumatoid arthritis, Sjögren’s syndrome, blistering skin disease, multiple sclerosis, and SLE (Amel Kashipaz et al., 2003; Iwata et al., 2011b). By contrast, one study found poor IL10 induction and regulatory function in human lupus when focusing on the CD24^high^CD38^high^ B cell pool (Blair et al., 2010). This discrepancy could either result from technical differences, patient cohort differences, or even a shift in excessive IL10 production by B cells of other phenotypes. Importantly, identifying which cells are responsible and when will be useful for biosignature phenotyping.

Antibody-secreting cell changes may also be informative for such profiling. Reports differ on whether increased functional and phenotypic ASC in SLE includes patients with mild disease (Wehr et al., 2004; Jacobi et al., 2010b; Korganow et al., 2010; Rodriguez-Bayona et al., 2010) or correlate with high disease activity (Odendahl et al., 2000; Anolik et al., 2004). The different outcomes could reflect biological disparities in patient groups, potentially through distinct contributions of autoantibody versus antibody-independent B cell functions (Huang et al., 2002; Anolik et al., 2004). It cannot be excluded that plasmablast expansion in SLE is a consequence, rather than a cause of systemic inflammation. Interestingly, HLA-DR^high^, but not HLA-DR^low^, plasmablast numbers correlate with SLE clinical manifestations (Jacobi et al., 2010b). Further defining subsets of ASC may contribute to eventual B cell profiling of lupus and other immunological conditions.

The wealth of information about human B cell subsets (Table 1) and their possible functions has been gathered in a fragmented fashion due to past constraints in resources and technology. We thus lack a comprehensive view of human B cell biology to adequately exploit for clinical benefit. However, further defining B cell subsets with high-dimensional flow cytometry and the appropriate data analysis methods could establish “signatures” or patterns of changes in B cell populations that may correlate with different clinical outcomes.

## Cytometry Approaches to Evaluate Human B Cells

Using our vast knowledge of B cell subsets to optimize disease diagnosis, prognosis, and treatment can only be accomplished through large cohort studies driven by state-of-the-art materials, analysis tools, and data management infrastructure (Harris et al., 2009; Maecker et al., 2012). Although many considerations described below are not unique to B cell cytometry, they are worth re-iterating due to the crucial importance of consistency in large studies. Consistency can be approached by performing as many steps as possible side-by-side, typically after shipping frozen cells collected at multiple clinical sites (or at multiple timepoints), then staining, and analyzing the samples together at only one central facility. For preparing, freezing, and thawing PBMC [by either conventional methods or by automated systems such as Smart Tube (Smart Tube Inc., Palo Alto, CA, USA)], having established protocols that are adhered to and documented as such allows backtracking problematic samples for critical decisions about inclusion of the data (Kaminski et al., 2012; Maecker et al., 2012). Importantly, awareness of which elements are most sensitive to processing allows one to circumvent the issues through compensatory study design. For example, surface CD62L expression is affected by Ficoll and by freezing (Maecker et al., 2012). Thus, we make it a point to find informative alternatives to such a marker when building our B cell cytometry panels for studies using frozen samples. Plasmablast and plasma cell detection is another challenge. Although functional ASC are still detectable by ELISpot after a freeze-thaw cycle, cells with only a surface marker-defined plasmablast phenotype have a significantly reduced recovery (Kyu et al., 2009). It is unknown if this effect is simply due to a loss of the respective cell-surface markers *per se* (in which case, the cells will be inaccurately accounted for in other populations) and/or if specific ASC subsets are affected differentially.

Building cytometry panels (either user-assembled or with pre-prepared lyophilized reagent cocktails, such as Lyoplates^™^ from BD Biosciences) should include critical consideration of monoclonal antibody clones and the appropriate conjugate fluorescent detection dyes, as well as maintaining and monitoring consistent reagent performance as has been elaborated elsewhere (Kaminski et al., 2012; Maecker et al., 2012). Also described elsewhere are instrument set-up considerations and the extent of controls needed, which are familiar to many flow cytometry users (Perfetto et al., 2006; Maecker et al., 2010, 2012; Chattopadhyay and Roederer, 2012), but become extremely important to maintain consistency in large studies. These elements include single-color instrument compensation controls, fluorescence-minus-one controls that help determine the lower-limits of a positive stain when gating, and monitoring and documenting instrument set-up and performance on different procedure days of a study. Monitoring instrument performance as well as staining consistency is also facilitated by having a designated biological sample that is aliquoted and frozen for staining with each run (Kaminski et al., 2012). The source of this control may either be a pool of healthy control PBMC or of hemochromatosis-patient PBMC, known to be highly enriched for B lymphocytes without excessive perturbances in known constituent subsets.

Ensuring consistent output is also achieved by establishing reagent panels in which the monoclonal antibody clones and their corresponding fluorescent conjugate detector dyes are identical for every iteration of the procedure. The Flow Immunophenotyping Technical Meeting at NIH (FITMaN) resulted in a proposed set of standards for developing such panels (Maecker et al., 2012). To ensure that due credit is attributed to the effort of panel development and for concise referencing, the journal *Cytometry A* established a specific publication mechanism: Optimized Multicolor Immunofluorescence Panel (OMIP; Roederer and Tarnok, 2010). As of August, 2012, there are 12 published OMIPs. Two of these panels encompass multiple hematopoietic lineages, one is for endothelial cells, one is for NK cells, and seven are for T cell subsetting, including antigen-binding cells and effector molecule detection. Clearly, this compilation of panels does not reflect the contribution of B cell subsets in human immune conditions as described above. To address this shortcoming, our group recently developed a 12-color panel to focus on memory-phenotype B cells (OMIP-003), although other subsets are detectable as well (Wei et al., 2011).

**Figure 1:**
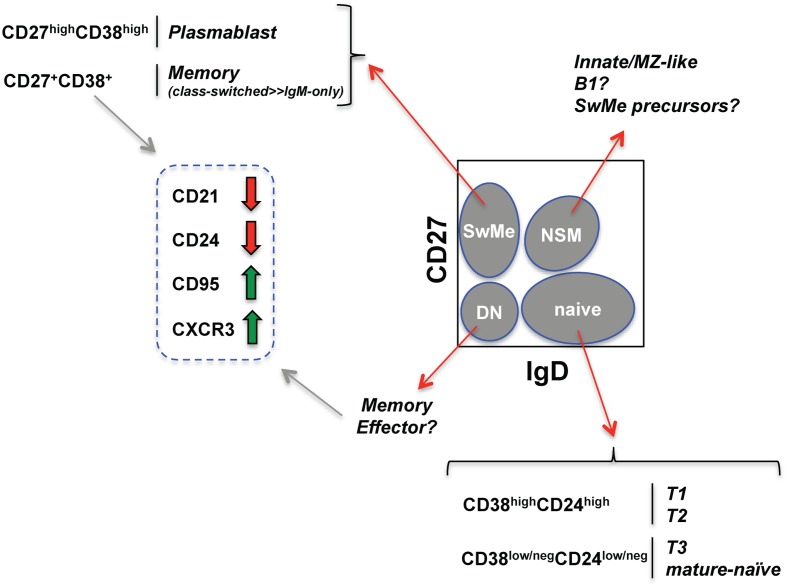
**Human B cell subsets identified with an established memory B cell fluorescent reagent panel (see Wei et al., 2011)**. Schematized flow cytometry plot (gated on viable PBMC CD19^+^ B cells) indicates four core B cell subsets (*gray* ovals) defined by CD27 and IgD expression. SwMe, switched memory; DN, double-negative; NSM, non-switched memory. The naïve core subset can be further subdivided into transitional and mature-naïve B cells. Distinguishing T3 from mature-naïve requires a mitochondrial dye extrusion step not included in the memory B cell panel. The switched memory and CD27^+/int^ memory-phenotype core subsets can be further evaluated for changes (*thick red and green arrows*) in the indicated markers known to be associated with B cell activation.

The OMIP-003 memory B cell panel (Table 2) includes IgD (more naïve) and CD27 (more differentiated) markers to derive four “core” or parent subsets described in previous sections (*gray ovals* in Figure 1). Within the parent IgD^+^CD27^neg^ naïve-phenotype fraction, displaying CD24 versus CD38 separates the T1/T2 transitional populations from T3/mature-naïve B cells as described above. Distinguishing T1 from T2 can be very difficult and is best approached by overlaying or comparative gate-drawing of a healthy bone marrow sample stained with the same panel, because bone marrow is highly enriched for T1 with the very highest levels of CD38 and CD24. T3 cannot be distinguished from mature-naïve with this memory B cell panel. Instead, an alternative panel that includes a reporter dye such as MTG is required (Palanichamy et al., 2009). The memory B cell panel also does not thoroughly elaborate previously investigated characteristics of the non-switched memory core subset, such as similarity to splenic marginal zone cells (Weller et al., 2004) or putative human B1 B cells, whose frequency in circulation is currently debated (Descatoire et al., 2011; Griffin et al., 2011; Perez-Andres et al., 2011).

**Table 2:** **Human memory B cell panel as per Wei et al. (2011)**.

Marker	Purpose/function
Aqua live/dead dye	Dead cell/debris exclusion
CD3	T cell exclusion
CD19	B lineage cell inclusion
IgD	Non-switched BCR isotype
CD27	Memory/activation
CD38	Differentiation
CD24	Differentiation
CD21	BCR co-receptor down-regulated upon activation
CD95	FAS death receptor up-regulated upon activation
CXCR3	Inflamed tissue homing receptor
B220 (CD45 isoform)	Differentiation/9G4^+^ Ab target autoantigen
9G4	BCR self-reactive idiotype

Within the remaining double-negative and switched memory core B cell subsets, CD21 loss, CD95 up-regulation, and CXCR3 up-regulation can be used to assess activation and propensity to home to inflamed tissue (Jacobi et al., 2008; Moir et al., 2008; Wei et al., 2011). Unlike in mice, B220 expression on human B cells is dynamic, being lost during GC differentiation. As a likely consequence, ∼60% of CD27^+^ memory-phenotype human B cells are normally B220^neg^ (Bleesing and Fleisher, 2003; Sanz et al., 2008). Unlike the CD27^+^ compartment in healthy subjects, the few remaining CD27^+^ B cells in patients with CD40L defects tend to be B220^+^ (Bleesing and Fleisher, 2003). This observation could indicate that the B220^+^CD27^+^ cells arise independently of GC, and/or of T cell help delivered by CD40L. Curiously, however, healthy-human CD27^+^ B cells expressing B220 have a greater propensity for CD21 loss and CD95 expression compared with the CD27^+^B220^neg^ cells (Morrow et al., 2008). It will be informative to compare expression of these various markers in human conditions including post-vaccination, infection, and immunodeficiency using advanced analysis techniques, as described below.

The anti-idiotype component of our memory B cell panel, rat anti-human monoclonal antibody clone 9G4, at once allows intrinsic detection of self-reactivity but also can complicate accurate measurement of B cells with the target self-reactive BCR. The 9G4 reagent detects human VH4.34-encoded immunoglobulin heavy-chain protein. Among the self-antigens detected by VH4.34 (9G4^+^) antibodies is human B220 (Cappione et al., 2004). Thus, circulating VH4.34 serum antibody bound to any B220^+^ B cell is detected by the rat anti-human reagent 9G4. This “painting” effect can be circumvented by incubating the cells in protein-containing media for 1 h at 37°C prior to staining with the 9G4 reagent (Cappione et al., 2004; Kobie et al., 2012).

Despite the numerous advantages of high-parameter fluorescence-based cytometry, developing and using 12- to 18-color panels is a significant challenge due to complications such as spectral overlap of the fluorescence signals, which becomes more problematic with increasing numbers of dyes. To circumvent such issues, DVD Sciences has developed Cytometry Time-of-Flight (CyTOF) analysis as an alternative method (Maecker et al., 2012). CyTOF mass cytometry detects cellular proteins bound by antibody (and other) reagents labeled with heavy isotopes of rare earth metals, whereas rhodium- and iridium-conjugated DNA intercalators are used for dead cell exclusion (Maecker et al., 2012). Besides nearly eliminating the need for channel compensation, the sensitivity of signals among isotopes is less variable than among fluorochromes (twofold versus 10- to 50-fold, respectively; Maecker et al., 2012). Thus far, 30 parameters have been simultaneously analyzed using this approach (Bendall et al., 2011; Maecker et al., 2012). However, with CyTOF, there is a threefold lower sampling efficiency and 25-fold lower flow rate (2 million cells/h) as well as lower staining indices than with fluorescence cytometry (Maecker et al., 2012). An additional limitation of CyTOF is the inability to recover viable or even intact cells because the mass cytometer needs to vaporize the cell for ionization (Maecker et al., 2012). Despite these current limitations that are impractical for very large numbers of samples, mass cytometry will likely develop into an advantageous tool allowing elaborate subsetting to be coupled with the detection of multiple indicators of intracellular signaling events such as protein phosphorylation (Bendall et al., 2011).

## Primary Cytometry Analysis

We previously described fluorescence cytometry event-gating strategies for our memory B cell panel using FlowJo software (TreeStar, Inc.; Wei et al., 2011; Kaminski et al., 2012), which, just as importantly as bench-level and instrumentation considerations, needs to be standardized for large studies. Critical considerations include the importance of: (1) excluding non-viable events and debris with a viability-discriminating dye, (2) excluding cell doublets and clumps using the various geometric dimensions of the light-scatter channels (FSC and SSC height versus width), and (3) choosing optimal scaling of plot axes to visualize and accurately gate all events, (4) focusing on the morphological scatter properties of the target parent population (i.e., lymphocytes; Herzenberg et al., 2006; Kaminski et al., 2012). In this latter regard, note that plasma cells and activated B cells can be large and have some granularity. Faithfully documenting gating strategies ensures consistency and facilitates the ability of other researchers to reproduce the results. An open-source R-package specification called Gating Markup Language (Gating-ML) aims to simplify such documentation and its exchange among users (Spidlen et al., 2008). In either case, gating, including what may be considered “pre-gating” steps to focus on the parental population (e.g., total B cells) should always be reported in figure legends and/or methods sections of publications. Citing published methods and OMIP-type articles can allow this description to remain concise.

Conventional manual gating of flow cytometry data, as described above, has served productively for many studies. However, this analysis approach has not kept pace with advances in fluorescent reagent development and instrumentation. Specific criticisms and short-comings of conventional manual gating include: (1) gated regions drawn by users may be unnatural and subjective boundaries that separate “populations” whose constituents may share unappreciated commonalities, (2) the iterative steps narrow how many events are available, and thus limit full utilization of high-parameter panels, and (3) user-to-user variation is inherent in such approaches. To begin addressing these issues, automated analysis methods are under development. One such approach is a density-based, model-independent algorithm called Flow Clustering without *k* (FLOCK; Qian et al., 2010), which is available through ImmPort (see text footnote 1) FLOCK identifies event clusters (populations) in cytometer data files (fcs files) based on simultaneous assessment of multiple fluorescent and scatter parameters. In this approach, the high-dimensional space formed by fluorescent parameter is partitioned with hyperbins, forming hyper-regions of events. Event-dense hyper-regions are merged with neighbors to form initial event clusters, followed by the calculation of a centroid for each event cluster. Euclidean distance between each event and the nearest centroid is used to assign the remaining events that are not in the event-dense hyper-regions to final clusters (populations). Using FLOCK, our group previously identified 17 event clusters of B lineage cells in healthy-subject PBMC stained with a 10-color fluorescent reagent panel (Qian et al., 2010). In the same study, three populations of plasmablast phenotype cells were identified whose post-vaccination kinetics very closely followed the functional ASC response, as detected by ELISpot assay.

**Figure 2:**
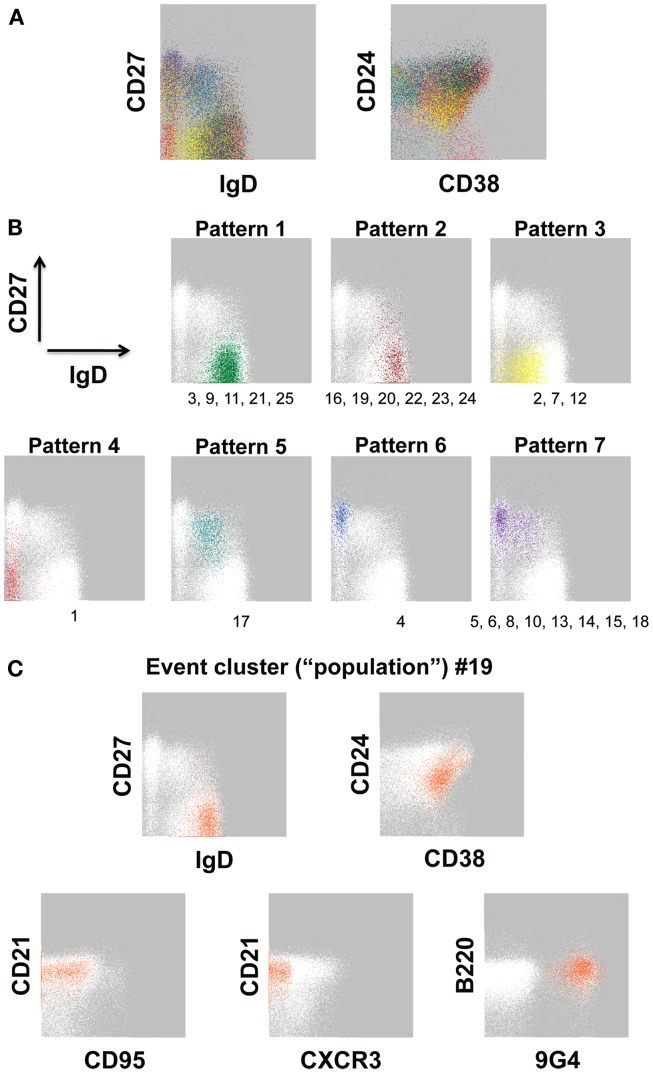
**Event clusters identified by FLOCK analysis**. FLOCK (Qian et al., 2010) version 1.0 was run unsupervised on human PBMC stained with the 12-color memory B cell reagent panel described in OMIP-003 (Wei et al., 2011) pre-gated on single, viable CD19^+^ lymphoid events. **(A)** Overlay of 25 populations, indicated in unique colors, identified in the sample displayed as two-dimensional plots. **(B)** Representative event clusters of the indicated Patterns based on CD27 versus IgD signals. Numbers below the plots indicate IDs of populations (see Table 3) with similar event distributions as displayed in the corresponding plot. **(C)** Population 19 displayed as all parameters analyzed by FLOCK. In **(B,C)**, *white* *events* on the *gray background* are the total CD19^+^ population, for reference. Characteristics of all 25 populations can be found in Table 3.

Using our 12-color panel (Wei et al., 2011), we more recently identified 25 B cell event clusters (populations, for simplicity; Figure 2A; Table 3) that we arbitrarily categorized into seven patterns according to IgD and CD27 expression based on conventionally defined subsets (Figure 2B). Whereas five of these clusters had a majority of their events in the IgD^+^CD27^neg^ region of the plot, six populations had a naïve-phenotype center plus several events also in the CD27^+^ region (Figure 2B, *Patterns 1 and 2*, respectively). Thus, based on simultaneous assessment of all fluorescent parameters in the analysis, these latter CD27^+^ events have more in common with their cluster-mates in the “naïve” area of the plot than they do with the other events conventionally assigned to the non-switched memory area of the plot. Putative biological relevance (e.g., common differentiation pathways) of these algorithm-defined associations remains to be validated. Notably, only one of the populations (*Pattern 5*) contained a majority of events in the non-switched memory area, accounting for 2.61% of the CD19^+^ parent population. Thus, the remaining 8.3% of the “non-switched memory” gate defined by conventional manual gating (not shown) of this particular sample may be more related to other subsets (e.g., those in the switched memory area, as in *Pattern 7*) than they are to each other. This outcome supports the concept that the IgD^+^CD27^+^ fraction may be a heterogeneous mixture of B cell subsets, as discussed above. Importantly, several established B cell subset characteristics were consistent in FLOCK analysis compared with conventional gating, as described earlier. This includes finding 9G4^+^ (likely autoreactive) B cells exclusively in naïve-phenotype clusters, and CD95 expression only in CD27^+^ clusters in the non-autoimmune subject analyzed here (Figure 2C; Table 3).

**Table 3:** **Characteristics of PBMC B cell clusters identified by FLOCK**.

	Popln	IgD	CD27	CD38	CD24	CD21	CD95	CXCR3	B220	9G4	% of CD19^+^
Pattern 1	3	+	−	+	+	+	−	−	+	−	12.80
	9	+	−	Low	Low	+	−	−	+	−	11.06
	11	+	−	+	+	+	−	−	+	−	6.41
	21	+	−	Low/−	+/Low	+/Low	−	−	+	−	5.51
	25	+	−	+	Low/−	−	−	−	+	+	2.04
Pattern 2	16	+	−/+	Low/−	+	+/Low	−	−	+	−	2.99
	19	+	−/+	High/+	+/Low	+	−	−	+	+	5.12
	20	+	−/+	+/Low	+/Low	+	−	−	+	−	7.29
	22	+	−/+	+	+	+/Low	−	±	+	+	2.95
	23	+	−/+	−	−	−	−	−	+	−	3.17
	24	+	−/+	Low/−	Low/−	−	−	−	+/Low	−	5.00
Pattern 3	2	Low	−	Low	Low	+	−	−	+	−	5.64
	7	Low	−	+	+	+	−	−	−	−	1.47
	12	Low	−	High	High	Low/−	−	−	+	−	2.13
Pattern 4	1	−	−/+	Spread	−	−	−	−	±	−	1.34
Pattern 5	17	+	+	Low/−	+	+	−	−	+/Low	−	2.61
Pattern 6	4	−	+	Low/−	Low/−	±	+	−	Low/−	−	1.56
Pattern 7	5	−/+	+	−	+	+	−	−	+/Low	−	2.74
	6	−/+	+	Low/−	+	+	−	−	+/Low	−	4.14
	8	−/+	+	−	+	+	−	−	+	−	2.31
	10	−/+	+	±	+	+/Low	−	±	−	−	1.49
	13	−/+	+	±	+	+	−	−	−	−	2.61
	14	−/+	+	+/Low	+	+	−	+	+/Low	−	2.05
	15	−/+	+	+/Low	+	+	−	−	Low	−	2.94
	18	−/+	+	±	±	±	±	+	+/Low	−	2.63

*FLOCK analysis of B cells was performed as described (Qian et al., 2010) with samples of non-autoimmune PBMC stained with the OMIP-003 memory B cell panel (Wei et al., 2011). Event cluster (popln) ID numbers and Pattern numbers correspond with Figure 2. Indicated are the marker expression levels for the cluster’s center*.

*The identical phenotypes of populations 3 and 11 may result from overpartitioning by FLOCK*.

*Note that the autoreactive idiotype 9G4 populations add-up to 10.1% and are found exclusively in naïve-phenotype (IgD^+^CD27^neg^) clusters, consistent with previous observations by manual gating of non-autoimmune subjects*.

The results in Figure 2 raise important questions about the potential B cell differentiation pathways from which our conventionally defined core peripheral B cell subsets derive. Are there some pathways in which CD27 is acquired before versus after loss of IgD, and *vice-versa*, if IgD is lost at all? Thus, an unsupervised FLOCK approach as demonstrated in Figure 2 can be used for hypothesis generation and exploratory profiling. In parallel, a supervised version of FLOCK is under development to incorporate user knowledge about cell populations and reagent panels to allow for a closer recapitulation of manual gating, if desired. In this way, researchers in a large study using the same reagent panel can establish the FLOCK settings desired, and then perform numerous cross-sample comparisons of hundreds of samples (in minutes per sample), which would otherwise be extremely labor-intensive by manual gating.

In addition to FLOCK, other event-analysis methods are under development, varying in clustering methods, whether a pre-determined number of clusters is used, modeling approaches, scalability, degree of supervision, etc. (Naim et al., 2010; Aghaeepour et al., 2012; Chattopadhyay and Roederer, 2012; Maecker et al., 2012). The SPADE (spanning-tree progression analysis of density-normalized events) algorithm goes a step further by mapping how the event cluster populations phenotypically relate to each other (Qiu et al., 2011). SPADE analysis produced a map of mouse bone marrow populations that recapitulated known hematopoietic differentiation stages of the cell phenotypes (Qiu et al., 2011). Another analysis using the flowMeans event clustering algorithm identified human peripheral T cell subset phenotype groups that were informative for predicting clinical progression to AIDS based on an analysis pipeline called FlowType (Chattopadhyay and Roederer, 2012). Given the various programs’ complexity and developmental status, determining which of the increasing number of available analysis algorithms would be most effective for a particular study may seem overwhelming to the immunological biologist. For this reason, the cytometry community, with support from the NIH, has invested in a series of evaluations called FlowCAP (Flow Cytometry: Critical Assessment of Population Identification Methods^2^). In this approach, identical data sets are evaluated by each algorithm in a series of Challenges to directly compare the programs, identify areas for improvement, and discuss possible standards for the cytometry analysis field. These efforts will facilitate the ability of the immunology research community to more fully take advantage of the numerous advances in B cell phenotyping described above.

## Management of Cytometry Data and Analytical Results

Regardless of the primary analysis method used, the enormous amounts of data generated by large multiparameter flow cytometry studies need to be managed efficiently together with clinical data for informative and non-redundant output. Housing and structuring the data in a relational database can facilitate such management. The Research Electronic Data Capture (REDCap) database developed at Vanderbilt University is an example of a comprehensive system for clinical data management (Harris et al., 2009). Options for downstream biological laboratory data management include LabKey (labkey.com) as well as Cytobank, a web-based resource modeled after gene expression data repositories in which scientists can perform comprehensive storage, organization, analysis, and sharing of flow cytometry data files (Kotecha et al., 2010). Once the data are further analyzed (described below) and ready for publication, their contributions in advancing scientific knowledge are best utilized if standards of adequate documentation are adhered to. MIFlowCyt (Minimum Information about a Flow Cytometry experiment) is a recommended standard for metadata to promote effective data set description for publication and to enhance reproducibility of analytical results. MIFlowCyt components are experiment overview, details about the specimen, data analysis details, and instrument details (Lee et al., 2008).

Finally, B cell phenotyping data need to be comprehensively analyzed to enable meaningful conclusions to be drawn from large human studies. Conventional graphing techniques are easily overwhelmed by the numbers of cell populations, the numbers of subjects and their treatments, and corresponding clinical read-outs involved in such studies. This issue can be addressed with more global profiling approaches in which all data are considered simultaneously, revealing system-wide views of immune-cell populations. Data clustering displayed in heat maps (color-coded sample-by-feature matrices, as are extensively used for gene expression analysis) offers many options for addressing experimental questions with a particular data set (D’Haeseleer, 2005). Hierarchical clustering seeks natural groupings of samples by partitioning data to minimize the distances (Euclidean distance, Pearson correlation, or Spearman correlation) among the samples within each group compared to distances among samples from different clusters (D’Haeseleer, 2005; Habib and Finn, 2006; Diaz-Romero et al., 2010; Blekherman et al., 2011). For example, hierarchical clustering with complete linkage and Pearson’s correlation as the distance metric identified 3–4 clusters of B-CLL patients based on the flow cytometry-defined characteristics of their circulating tumor cells (Habib and Finn, 2006). Further analysis of this data showed that patients in a cluster characterized by CD38^low^ B lineage tumor cells correlated with longer patient survival (Habib and Finn, 2006). Similarly, clustering organizes the populations so that those that vary in a similar fashion over the set of samples are grouped together. In SLE, *k*-means clustering, an alternative approach with a pre-specified number of clusters, based on Euclidean distance among clinical and demographic features, identified three patient groups, one of which correlated with a high mortality rate (To et al., 2009). It will be advantageous to combine these types of informative analyses with the above-described advances in extensive phenotypic B cell subsetting to identify clinical-associated signatures in multiple areas, including infection and autoimmunity prognosis, vaccine responses, and transplant tolerance.

Displaying clustered data as heat maps may suffice for addressing the experimental question of a particular study. However, in some cases, a related and complementary data exploration approach, Principal Component Analysis (PCA) can elucidate how the variation of analyzed cell populations (variables) informs us about clinically useful relationships among the data. PCA is a dimension-reduction technique that expresses the original data as a set of new, transformed variables that are linear combinations of the original variables. Because many of the original variables are likely to be correlated across a set of samples, a large fraction of the total variance in the data set is captured in the first few so-called principal components (Quackenbush, 2001; Misra et al., 2002). In this way, the high dimensionality of the data is reduced into a form that is more easily visualized and comprehended (Blekherman et al., 2011). Note that PCA is unsupervised in that no information about sample group membership is supplied and that most of the variation in a data set, captured by the first two principal components may be due to process variability or noise, and not the result of a biological or clinical cause. Nonetheless, by superimposing sample group membership onto samples in a PCA plot, segregation of groups can be revealed. For example, PCA (as well as data clustering) on flow cytometry-derived data has shown T cell subsets that are higher in centenarians compared with younger individuals (Lugli et al., 2007).

## Concluding Remarks

The phenotypic diversity of human B cell subsets provides an opportunity to interrogate B profiling to revolutionize immunological disease diagnosis and optimize treatment modalities, including, but not limited to, B cell-targeted therapies. Coupling cytometry-based phenotypic B cell analysis with cell morphology, immunoglobulin gene analysis (V region usage and somatic hypermutation patterns), functional *in vitro* assays (including cytokine and antibody secretion), and profiling after B cell-targeted therapies can also further elucidate the mechanisms driving such diseases, paving the way for more optimized therapies, as well as understanding normal B cell biology, which can be harnessed for improved vaccine development. Importantly, the limitations once thought to restrict human immunology to pauci-informative descriptive analysis can be overcome with improvements in flow cytometry *per se*, as well as developing advances in flow data analysis and data management infrastructure.

## Conflict of Interest Statement

The authors declare that the research was conducted in the absence of any commercial or financial relationships that could be construed as a potential conflict of interest.

## Footnotes

^1^immport.niaid.nih.gov

^2^flowcap.flowsite.org
